# Growth in Children with Autosomal Recessive Polycystic Kidney Disease in the CKiD Cohort Study

**DOI:** 10.3389/fped.2016.00082

**Published:** 2016-08-10

**Authors:** Erum A. Hartung, Katherine M. Dell, Matthew Matheson, Bradley A. Warady, Susan L. Furth

**Affiliations:** ^1^Division of Nephrology, Department of Pediatrics, Children’s Hospital of Philadelphia, Perelman School of Medicine, University of Pennsylvania, Philadelphia, PA, USA; ^2^Department of Pediatrics, Center for Pediatric Nephrology, Cleveland Clinic Children’s, Case Western Reserve University, Cleveland, OH, USA; ^3^Department of Epidemiology, Johns Hopkins University, Baltimore, MD, USA; ^4^Division of Pediatric Nephrology, Children’s Mercy Hospital, Kansas City, MO, USA

**Keywords:** growth, autosomal recessive polycystic kidney disease, children, growth hormone

## Abstract

**Background:**

Previous studies have suggested that some children with autosomal recessive polycystic kidney disease (ARPKD) have growth impairment out of proportion to their degree of chronic kidney disease (CKD). The objective of this study was to systematically compare growth parameters in children with ARPKD to those with other congenital causes of CKD in the chronic kidney disease in Children (CKiD) prospective cohort study.

**Methods:**

Height SD scores (*z*-scores), proportion of children with severe short stature (*z*-score < −1.88), rates of growth hormone use, and annual change in height *z*-score were analyzed in children with ARPKD (*n* = 22) compared with two matched control groups: children with aplastic/hypoplastic/dysplastic kidneys (*n* = 44) and obstructive uropathy (OU) (*n* = 44). Differences in baseline characteristics were tested by Wilcoxon rank-sum test or Fisher’s exact test. Matched differences in annual change in height *z*-score were tested by Wilcoxon signed-rank test.

**Results:**

Median height *z*-score in children with ARPKD was −1.1 [interquartile range −1.5, −0.2]; 14% of the ARPKD group had height *z*-score < −1.88, and 18% were using growth hormone. There were no significant differences in median height *z*-score, proportion with height *z*-score < −1.88, growth hormone use, or annual change in height *z*-score between the ARPKD and control groups.

**Conclusion:**

Children with ARPKD and mild-to-moderate CKD in the CKiD cohort have a high prevalence of growth abnormalities, but these are similar to children with other congenital causes of CKD. This study does not support a disease-specific effect of ARPKD on growth, at least in the subset of children with mild-to-moderate CKD.

## Introduction

Autosomal recessive polycystic kidney disease (ARPKD) is an inherited renal cystic disease that is typically diagnosed in infancy and leads to progressive chronic kidney disease (CKD) (1). Previous studies have reported growth impairment in approximately 25% of children with ARPKD (2, 3). In a subset of patients in prior reports, growth failure appeared to precede a significant deterioration in glomerular filtration rate (GFR) and could not be readily attributed to other comorbidities, such as metabolic acidosis, anemia, primary growth hormone deficiency, malnutrition, liver dysfunction, or chronic lung disease (3, 4). Similar observations have been made in a mouse model of ARPKD, in which a subset of mice had growth impairment prior to the onset of significant renal disease (5). Some authors have speculated that ARPKD may have effects on growth independent of decreased kidney function, perhaps due to disturbances in the growth hormone/insulin-like growth factor (IGF)-1 axis or *via* decreased expression of growth hormone receptors in the liver (4). However, other authors have observed that growth impairment in patients with ARPKD does appear to correlate with decreased kidney function (2, 6).

The objective of this study was to characterize height deficits and linear growth in patients with ARPKD enrolled in the chronic kidney disease in children (CKiD) study, a prospective, longitudinal investigation of children with mild-to-moderate CKD due to a wide range of diagnoses. We sought to test the hypothesis that children with ARPKD will have growth impairment out of proportion to their degree of kidney dysfunction, by comparing growth in children with ARPKD to those with other congenital causes of CKD.

## Materials and Methods

### Study Participants

Autosomal recessive polycystic kidney disease subjects and controls were selected from among those enrolled in CKiD, a longitudinal, prospective study of children with mild-to-moderate CKD. Complete eligibility criteria for CKiD have been published in detail elsewhere (7) and include age 1–16 years, estimated GFR of 30–90 mL/min/1.73 m^2^ calculated using the original Schwartz formula (8, 9) at enrollment, and absence of severe syndromic disease. The CKiD study protocol was approved by the Institutional Review Boards at all participating sites, and informed consent was obtained from all caregivers.

The current study was performed as a control-matched analysis within the CKiD cohort. All children enrolled in CKiD with a diagnosis of ARPKD were included in this analysis. Controls were obtained from two diagnostic groups with other congenital renal diseases: (1) aplastic/hypoplastic/dysplastic (A/H/D) kidneys and (2) obstructive uropathy (OU). Controls were matched to ARPKD subjects in a 2:1 ratio based on baseline ieGFR [GFR measured by plasma disappearance of iohexol (iGFR) or eGFR (10), if iGFR not available], age at study entry, and age at diagnosis. Matching was performed by calculating the Euclidean distance in 3-dimensional space (with baseline ieGFR, age at study entry, and age at diagnosis being the dimensions) between each of the 22 ARPKD subjects and each potential control (all subjects in CKiD with A/H/D or OU diagnoses, with each control group matched separately). The smallest distance (closest match) was then selected out of all possible matches; that control was removed, and then the procedure was repeated to choose closest matches until each ARPKD subject had two distinct matches.

### Growth Parameters

Height or length was determined at all CKiD study visits as the mean of two independent measurements, using a stadiometer for children >2 years old who are able to stand, or using a firm box in the supine position for children unable to stand (7). Age- and sex-specific height SD scores (*z*-scores) were calculated using United States (US) Centers for Disease Control and Prevention (CDC) growth charts of normative data (11). Growth hormone usage was ascertained at each visit using a standardized medication questionnaire. GFR was estimated yearly using the updated biomarker-based Schwartz formula (eGFR) (10) and was measured every other year using plasma disappearance of iohexol (iGFR) (12).

The primary outcomes examined in this study were height *z*-score, proportion of children with severe short stature (height *z*-score < −1.88), rates of growth hormone use at baseline, and annual change in height *z*-score. We also examined other covariates that could have an influence on linear growth, including weight and body mass index (BMI, as indicators of nutritional status), pubertal (Tanner) stage, serum bicarbonate concentrations, and use of bicarbonate supplementation [to evaluate degree of metabolic acidosis, which previous studies have shown can affect linear growth (13)], intact parathyroid hormone (iPTH) concentrations and use of phosphate binders (to assess for secondary hyperparathyroidism), hemoglobin [some studies have identified anemia as a risk factor for poor growth in CKD (14)], platelet count [to assess severity of portal hypertension in children with ARPKD (15)], albumin (to assess liver synthetic function), and need for intensive care unit and days of hospitalization at birth (to assess for severity of other comorbidities such as pulmonary hypoplasia). Additional data regarding neonatal course, other comorbidities, growth hormone levels, or IGF binding protein levels were not available in this cohort. As an exploratory analysis, we also compared annual change in height *z*-score between users and non-users of growth hormone in all three groups.

### Statistical Analysis

Demographic and clinical characteristics were reported as median (interquartile range, IQR) or number, *n* (%), for each group. Differences between the ARPKD group and each control group were tested by Wilcoxon rank-sum test or Fisher’s exact test. Annualized change in height *z*-score was calculated using individual regressions for each subject incorporating all available follow-up measurements. Matched differences were calculated as the difference between an ARPKD subject’s value and the average value of the two matched controls, and these distributions were tested for difference from 0 by Wilcoxon signed-rank test.

Since the sample size of ARPKD patients enrolled in CKiD was fixed, we did not perform power calculations to determine sample size. However, given the available sample size (*n* = 66 total, with *n* = 22 ARPKD and *n* = 44 total controls), we would have been able to detect a difference in baseline height *z*-score of 0.83 or a difference in annual change in height *z*-score slope of 0.16.

## Results

### Demographic and Clinical Features

The baseline demographic and clinical features of the 22 ARPKD subjects and the A/H/D and OU control groups (*n* = 44 each) have been reported in our previous publication examining kidney disease progression in ARPKD subjects in the CKiD cohort (16). The ARPKD and control groups (A/H/D and OU) were successfully matched on baseline ieGFR, age at study entry, and age at diagnosis. For the ARPKD cohort, the mean age at study entry was 7.9 years and the mean baseline ieGFR was 43 mL/min/1.73 m^2^. Twenty-seven percent of ARPKD patients were premature (<36 weeks gestation), and 18% had low birth weight (<2500 g). There were no significant differences between the ARPKD subjects and the two matched control groups for any of these parameters.

### Growth Parameters and Other Covariates at Baseline

Baseline growth characteristics of the ARPKD group and the A/H/D and OU control groups are shown in Table 1. All three groups had height *z*-scores below the normal range compared with normative data for US children, but there were no significant differences in median height *z*-score among the three groups (ARPKD −1.1 vs. A/H/D −0.8, *p* = 0.97, ARPKD −1.1 vs. OU −0.6, *p* = 0.63). A large proportion of children in all three groups had severe short stature (height *z*-score less than −1.88, i.e., <3rd percentile), but there were no significant differences between the three groups (ARPKD 14% vs. A/H/D 19%, *p* = 0.74, ARPKD 14% vs. OU 23% *p* = 0.52). The proportions of children using growth hormone were also similar between the three groups (ARPKD 18% vs. A/H/D 9%, *p* = 0.42, ARPKD 18% vs. OU 14%, *p* = 0.72).

**Table 1:** **Demographic, clinical, and growth characteristics of autosomal recessive polycystic kidney disease (ARPKD) group compared to control groups with renal aplasia/hypoplasia/dysplasia (A/H/D) or obstructive uropathy (OU)**.

Characteristic	ARPKD subjects (*n* = 22)	A/H/D controls (*n* = 44)	*p*	OU controls (*n* = 44)	*p*
**Baseline characteristics**					
Tanner stage I	19 (90%)	35 (85%)	0.71	40 (91%)	>0.99
Height *z*-score^a^	−1.1 [−1.5, −0.2]	−0.8 [−1.7, 0.1]	0.97	−0.6 [−1.7, 0.3]	0.63
Height *z*-score < −1.88^b^	3 (14%)	8 (19%)	0.74	10 (23%)	0.52
Growth hormone use	4 (18%)	4 (9%)	0.42	6 (14%)	0.72
Weight *z*-score^a^	−0.2 [−1.0, 0.6]	−0.3 [−1.2, 0.9]	0.85	−0.2 [−1.2, 0.7]	0.87
BMI *z*-score	0.4 [−0.1, 1.1]	0.2 [−0.5, 1.2]	0.70	0.5 [−0.1, 1.2]	0.85
Serum bicarbonate (mEq/L)	22 [20, 23]	23 [20, 25]	0.28	22 [20, 24]	0.70
Receiving bicarbonate supplementation^b^	3 (14%)	7 (16%)	>0.99	4 (9%)	0.68
iPTH (pg/mL)	45 [30, 72]	49 [31, 99]	0.59	62 [29, 68]	0.47
Receiving phosphate binder^b^	5 (23%)	9 (20%)	>0.99	8 (18%)	0.75
Hemoglobin (g/dL)^a^	11.7 [10.8, 12.4]	12.6 [12.0, 13.5]	0.001	12.6 [12.0, 13.7]	0.001
Platelets (×10^3^/μL)	254 [149, 346]	274 [218, 320]	0.21	279 [232, 332]	0.14
Albumin (g/dL)	4.5 [4.4, 4.6]	4.4 [4.2, 4.6]	0.38	4.4 [4.3, 4.6]	0.24
ICU at birth^b^	10 (45%)	28 (64%)	0.19	23 (53%)	0.61
Days in hospital at birth^a^	3 [2, 21]	7 [3, 21]	0.24	5 [2, 30]	0.76
**Longitudinal characteristics**					
Annual change in height *z*-score			
Among GH users^c^	−0.038	0.097	^d^	0.034	^d^
Among GH non-users^a^	−0.023	−0.028		−0.008
	[−0.151, 0.094]	[−0.116, 0.079]		[−0.098, 0.080]

*A/H/D, renal aplasia/hypoplasia/dysplasia; ARPKD, autosomal recessive polycystic kidney disease; BMI, body mass index; GH, growth hormone; iPTH, intact parathyroid hormone; OU, obstructive uropathy*.

*^a^Median [interquartile range]*.

*^b^Number (% of total)*.

*^c^Median (insufficient data to calculate interquartile range)*.

*^d^Sample size too small for statistical comparison*.

Examination of other covariates showed no significant differences between the ARPKD group and the A/H/D and OU control groups in weight and BMI *z*-scores, pubertal (Tanner) stage, serum bicarbonate concentrations, use of bicarbonate supplementation, iPTH concentrations, and use of phosphate binders. The ARPKD group had lower median hemoglobin than the A/H/D and OU control groups (ARPKD 11.7 g/dL vs. A/H/D 12.6 g/dL, *p* = 0.001, ARPKD 11.7 g/dL vs. OU 12.6 g/dL, *p* = 0.001) (Table 1).

We assessed for severity of portal hypertension in the ARPKD group by comparing platelet counts across the groups. Although there were no significant differences in median platelet counts between the ARPKD and A/H/D and OU control groups (Table 1), a subset of the ARPKD group (6 out of 22, 27%) did have clinical evidence of portal hypertension, as evidenced by thrombocytopenia (platelet count < 150,000/μL). Median height *z*-score at baseline in ARPKD patients with thrombocytopenia (*n* = 6) was −0.39 [IQR −1.47, −0.16], compared with −1.28 [IQR −1.48, −0.27] in those with normal platelet counts (*n* = 16). Although the small number of subjects precludes statistical analysis, the similarity of the interquartile ranges in the two groups suggests that there was no significant effect of portal hypertension on baseline height. Serum albumin was similar between the ARPKD and control groups, indicating normal liver synthetic function (Table 1).

Although detailed clinical information regarding the presence of pulmonary hypoplasia at birth was not available, the ARPKD group had similar prevalence of needing ICU care at birth and similar durations of hospitalization compared to the A/H/D and OU control groups (Table 1).

### Longitudinal Growth Rates

**Figure 1:**
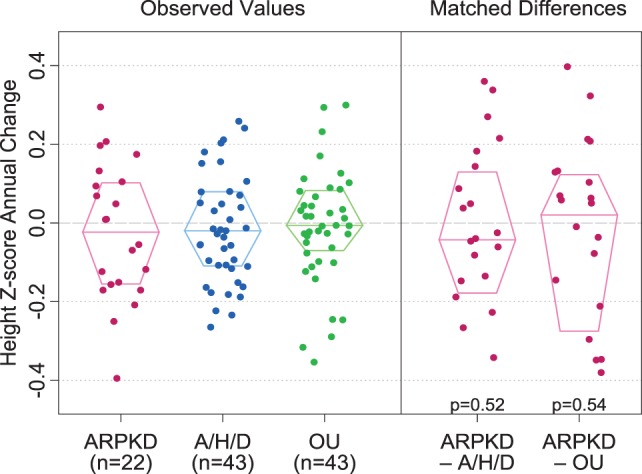
**Annual change in height *z*-scores in children with autosomal recessive polycystic kidney disease (ARPKD), aplastic/hypoplastic/dysplastic kidneys (A/H/D), and obstructive uropathy (OU)**. Observed values are shown on the left and matched differences between the ARPKD group and each control group are shown on the right. *p* Values for matched differences between ARPKD and each control group are as shown. Boxes display the median and interquartile range of the observations.

Observed values for annual change in height *z*-score were −0.02 [IQR −0.16, 0.10] in the ARPKD group, −0.02 [−0.11, 0.08] in the A/H/D group, and −0.01 [−0.07, 0.08] in the OU group (Figure 1). Examination of matched differences in annual change in height *z*-score showed no significant differences between the ARPKD and A/H/D groups (*p* = 0.52) or between the ARPKD and OU groups (*p* = 0.54).

We also explored the response to growth hormone in the ARPKD and control groups by examining annual change in height *z*-score in users and non-users of growth hormone. Although the small sample size precludes statistical analysis, it is interesting to note that the ARPKD subjects who were receiving growth hormone (*n* = 4) at baseline had lower annual change in height *z*-score (median −0.038) compared with those with A/H/D (*n* = 4, median 0.097) or OU (*n* = 6, median 0.034) (Table 1).

## Discussion

In this study, we sought to determine whether children with ARPKD have deficits in height and linear growth beyond those attributable to decreased kidney function. We compared growth parameters in a well-characterized cohort of children with ARPKD in the CKiD cohort study to matched controls in two diagnostic groups, A/H/D and OU. These groups were chosen because they are also predominantly tubulointerstitial disorders and have a similar age of presentation as ARPKD. In a previous analysis, we have also shown that these two control groups have similar rates of GFR decline to children with ARPKD (16).

We found that children with ARPKD had a high prevalence of growth disorders, but the findings were overall similar to what was seen in children with other congenital causes of CKD. The ARPKD group had similar height *z*-scores, proportion of children with severe short stature, and rates of growth hormone use at baseline and had similar rates of annual change of height *z*-score compared to children with A/H/D or OU. Height, weight, and BMI *z*-scores in all three groups were also comparable to those reported in children with non-glomerular disorders in the CKiD cohort as a whole (13). Body weight, BMI, and pubertal stage were similar among the three groups. All three groups had serum bicarbonate concentrations and iPTH within the normal range, with similar usage rates of bicarbonate supplementation and phosphate binders, indicating no significant metabolic acidosis or hyperparathyroidism.

The median platelet count in the ARPKD group was within the normal range, indicating overall low severity of portal hypertension in this group. Given the relatively mild liver phenotype in this group, we may not have been able to detect differences in growth attributable to portal hypertension. However, the similarities in baseline height *z*-scores between ARPKD patients with vs. without thrombocytopenia were not suggestive of a strong effect of portal hypertension on growth in this cohort.

Overall, these data suggest that there is no disease-specific effect of ARPKD on linear growth beyond that attributable to CKD. As this is the first study to systematically compare ARPKD children with cohorts of patients with other congenital causes of CKD, it is likely that earlier observations of significant growth failure in children with ARPKD were attributable to other risk factors, such as early onset CKD, poor nutrition, prematurity, or other comorbidities. CKD during infancy and early childhood is a known risk factor for growth failure (17, 18), and many children with ARPKD present early in infancy. Much of the risk of growth failure in infants with CKD is due to inadequate nutrition (19), which children with ARPKD may be at particular risk for due to enlarged kidneys that can interfere with enteral feeding. Children with CKD who have abnormal birth history, including low birth weight, prematurity, and need for intensive care unit (ICU), are also at higher risk for short stature (20). A substantial proportion of children with ARPKD are born prematurely (27% in the current study), and many require ICU care and mechanical ventilation due to pulmonary hypoplasia (3), which may contribute further to their risk of growth failure.

Given prior reports showing relative under-utilization of growth hormone in children with CKD (13, 21), it is encouraging to note the relatively high rate of growth hormone use in this ARPKD cohort: 4 out of the 22 children (18%) reported growth hormone use, which actually exceeded the number of children with severe short stature at baseline [3 out of 22 (14%) with height *z*-score < −1.88]. As an exploratory analysis, we compared linear growth in children who were receiving growth hormone compared to those who were not. Although this analysis was suggestive of a worse response to growth hormone in the ARPKD group compared with the A/H/D and OU control groups, our sample size was inadequate to draw any conclusions regarding response to growth hormone in ARPKD. A previous study has shown that children with ARPKD and short stature respond well to growth hormone therapy (4). Therefore, we believe children with ARPKD and growth failure should be offered growth hormone therapy in accordance with published guidelines for children with CKD (22).

Strengths of this study include accurate, standardized evaluations of height, along with detailed clinical characterization of many covariates relevant for growth. This is also the first study to systematically compare linear growth in children with ARPKD to those with other causes of CKD, which allowed us to examine disease-specific effects of ARPKD on growth. The availability of longitudinal data also allowed us to compare linear growth rates over time. Limitations of this study include a relatively small sample size and the inclusion in CKiD of only children with mild-to-moderate CKD. Thus, this population may not be representative of children with ARPKD as a whole. Despite the small sample size, we believe this is the largest cohort of ARPKD patients to undergo detailed characterization of growth with longitudinal measures. Therefore, description of growth parameters in this cohort adds to the limited body of literature on growth failure in children with ARPKD.

In conclusion, this study shows that children with ARPKD and mild-to-moderate CKD have a high prevalence of growth abnormalities, but these are comparable to children with other causes of CKD. Clinicians should, therefore, monitor linear growth closely in children with ARPKD and other causes of congenital CKD and should utilize strategies to improve growth, including ensuring adequate enteral nutrition and normal acid–base status and prescribing growth hormone as indicated.

## Author Contributions

EH wrote the first draft of the manuscript. KD, EH, BW, and SF designed the study and interpreted the data. MM analyzed and interpreted the data. All authors critically revised the manuscript and approved of the final version to be published.

## Conflict of Interest Statement

The authors declare that the research was conducted in the absence of any commercial or financial relationships that could be construed as a potential conflict of interest.
